# Correction: Preterm birth and neonatal mortality in selected slums in and around Dhaka City of Bangladesh: A cohort study

**DOI:** 10.1371/journal.pone.0321669

**Published:** 2025-04-02

**Authors:** Abdur Razzaque, Anisur Rahman, Razib Chowdhury, A. H. M. Golam Mustafa, Shakera Naima, Farzana Begum, Sohana Shafique, Bidhan Krishna Sarker, Mohammad Zahirul Islam, Minjoon Kim, Margub Aref Jahangir, Ziaul Matin, Jannatul Ferdous, Maya Vandenent, Daniel D. Reidpath

The frequency of Survive (N = 6724) for Operation under Mode of delivery in Table 1 is incorrect. It should have been 1903 (98.3%). Please see the correct Table 1 below.

**Table 1:** Percent distribution of total sample.

Sociodemographic characteristics	Total (N = 6989)	Survive (N = 6724)	Death (N = 265)
**Mother’s age (years)**			
<18	608 (8.7%)	576 (94.7%)	32 (5.3%)
18-24	3816 (54.6%)	3666 (96.1%)	150 (3.9%)
25 or more	2565 (36.7%)	2482 (96.8%)	83 (3.2%)
**Years of schooling of mother**			
None	1962 (28.1%)	1877 (95.7%)	85 (4.3%)
1-4	1510 (21.6%)	1446 (95.8%)	64 (4.2%)
5 or more	3517 (50.3%)	3401 (96.7%)	116 (3.3%)
**Mother’s occupation**			
Not working	5148 (73.7%)	4950 (96.1%)	198 (3.9%)
Working	1841 (26.3%)	1774 (96.4%)	67 (3.6%)
**Sex of Child**			
Male	3580 (51.2%)	3427 (95.7%)	153 (4.3%)
Female	3409 (48.8%)	3297 (96.7%)	112 (3.3%)
**Mode of delivery**			
Normal	5053 (72.3%)	4821 (95.4%)	232 (4.6%)
Operation	1936 (27.7%)	1903 (98.3%)	33 (1.7%)
**Birth attendant**			
Skilled	2536 (36.3%)	2471 (97.4%)	65 (2.6%)
Unskilled	4453 (63.7%)	4253 (95.5%)	200 (4.5%)
**Place of delivery**			
Home	3399 (48.6%)	3254 (95.7%)	145 (4.3%)
Hospital	3590 (51.4%)	3470 (96.7%)	120 (3.3%)
**ANC visits**			
No visits	1117 (16.0%)	1070 (95.8%)	47 (4.2%)
1-3 visits	3301 (47.2%)	3151 (95.5%)	150 (4.5%)
4 or more visits	2571 (36.8%)	2503 (97.4%)	68 (2.6%)
**Preterm birth**			
Very preterm	151 (2.2%)	119 (78.8%)	32 (21.2%)
Moderate preterm	268 (3.8%)	240 (89.6%)	28 (10.4%)
Late preterm	1097 (15.7%)	1052 (95.9%)	45 (4.1%)
Term birth	5473 (78.3%)	5313 (97.1%)	160 (2.9%)

In Table 3, the frequency and the percentages under the ‘No. of birth (percent)’ column are incorrect. Please see the correct Table 3 below.

**Table 3:** Number of births, number of deaths, neonatal death rates, relative risks, and population attributable fractions by gestation age categories.

Gestation age categories^+^	No. of birth (percent)	No. of death (percent)	NMR (per 1000 livebirths)	RR (95% CI)	PAF (95% CI)
Very preterm	151 (2.16)	32 (12.08)	209.15	7.15 (5.07-10.08)	0.14 (0.15- 0.28)
Moderate preterm	268 (3.83)	28 (10.57)	105.25	3.60 (2.45-5.27)	0.10 (0.07-0.14)
Late preterm	1097 (15.70)	45 (16.98)	40.98	1.40 (1.01-1.93)	0.06 (0.03-0.05)
Preterm	1516 (21.69)	105 (39.63)	69.21	2.37 (1.87-3.01)	0.23 (0.18-0.43)
Term	5473 (78.31)	160 (60.38)	29.23	1.00	–
All	6989	265	37.92	–	–

Note: CI = Confidence interval; NMR = Neonatal Mortality Rate; RR = Relative risk; PAF = Population Attributable Fraction;

+ Preterm birth (28 and 36 weeks): Very preterm (28 to 31 weeks), moderate preterm (32 to 33 weeks), and late preterm (34 to 36 weeks); Term birth (37 or more weeks).
