# Supplementary material for: Preterm birth and neonatal mortality in selected slums in and around Dhaka City of Bangladesh: A cohort study
Source: PLoS One. 2024 Jan 19;19(1):e0284005. doi: 10.1371/journal.pone.0284005 (PMC10798464; doi:10.1371/journal.pone.0284005)
Supplement: S1 Checklist — (DOCX) [file pone.0284005.s001.docx]

STROBE Statement—checklist of items that should be included in reports of observational studies

|  | Item No. | | Recommendation | | Page  No. | | | Relevant text from manuscript | | | |
| --- | --- | --- | --- | --- | --- | --- | --- | --- | --- | --- | --- |
| **Title and abstract** | 1 | | (*a*) Indicate the study’s design with a commonly used term in the title or the abstract | | 1 | | | In the title of the manuscript. | | | |
|  |  |  | (*b*) Provide in the abstract an informative and balanced summary of what was done and what was found | | 2-3 | | | Although under-five mortality declined in Bangladesh……\|\| …. The study revealed that preterm babies ……….. 6% for late preterm. | | | |
| Introduction | | | | | | |  | | | |  |
| Background/rationale | 2 | | Explain the scientific background and rationale for the investigation being reported | | 4-5 | | | The survival chance of a baby depends on gestation age…….. increasing in some developed countries. Preterm birth complications are estimated to be responsible for 35%.....neonatal mortality significantly [20-26]. | | | |
| Objectives | 3 | | State specific objectives, including any prespecified hypotheses | | 6 | | | The objective of the study is to assess the level and determinants of preterm birth and the contribution of preterm birth to neonatal mortality using data from selected slums in and around Dhaka city. | | | |
| Methods | | | | | | |  | | | |  |
| Study design | 4 | | Present key elements of study design early in the paper | | 6 | | | The study used data from urban Health and Demographic Surveillance System (HDSS) which operates on some purposively selected *slums* from Dhaka (North & South) and Gazipur City Corporations, where icddr,b has been maintaining the HDSS since 2015 (for detail see *Slum Health in Bangladesh [29]*). We extracted data of a birth cohort from urban HDSS and followed for <29 days since birth. | | | |
| Setting | 5 | | Describe the setting, locations, and relevant dates, including periods of recruitment, exposure, follow-up, and data collection | | 6-7 | | | The study used data from urban Health and Demographic Surveillance System (urban HDSS) which operates on some purposively selected *….. \|\|* All the inhabitants of those selected slums were interviewed for baseline census in 2015 covering 118,238 population (3.8 persons per households….. | | | |
| Participants | 6 | | (*a*) *Cohort study*—Give the eligibility criteria, and the sources and methods of selection of participants. Describe methods of follow-up  *Case-control study*—Give the eligibility criteria, and the sources and methods of case ascertainment and control selection. Give the rationale for the choice of cases and controls  *Cross-sectional study*—Give the eligibility criteria, and the sources and methods of selection of participants | | 6 | | | For each livebirth, data were collected on the date of delivery, place of delivery, details on maternity care (antenatal, delivery, and postnatal practice). In this study, all the livebirths in the study area for the period 2016-2018 were included and followed for survival up to the neonatal period. | | | |
|  |  |  | (*b*) *Cohort study*—For matched studies, give matching criteria and number of exposed and unexposed  *Case-control study*—For matched studies, give matching criteria and the number of controls per case | | NR | | |  | | | |
| Variables | 7 | | Clearly define all outcomes, exposures, predictors, potential confounders, and effect modifiers. Give diagnostic criteria, if applicable | | 7-8 | | | Gestation age was estimated by subtracting the date of delivery and the date of LMP which later converted into gestation weeks. Those born between 28 and 36 weeks of gestation were termed as preterm birth, and sub-categories were very preterm (28 to 31 weeks), moderate preterm (32 to 33 weeks), and late preterm (34 to 36 weeks); those born at 37 or.….  Women’s age at birth, education, working status, sex of the newborn, number of antenatal visits, and delivery related factors- mode, place and attendants were adjusted in logistic regression analyses (Model-II). | | | |
| Data sources/ measurement | 8* | | For each variable of interest, give sources of data and details of methods of assessment (measurement). Describe comparability of assessment methods if there is more than one group | | 7 | | | Those born between 28 and 36 weeks of gestation were termed as preterm birth, and sub-categories were very preterm (28 to 31 weeks), moderate preterm (32 to 33 weeks), and late preterm (34 to 36 weeks); those born at 37 or more weeks of gestation were classified as term birth.  ….while ‘40 weeks’ was used as reference; while for categories of gestation age, term births (37 to 42 weeks) used as a reference category | | | |
| Bias | 9 | | Describe any efforts to address potential sources of bias | | 6 | | | Once the conception was confirmed, the woman was followed for subsequent pregnancy outcomes (livebirth, stillbirth, induce miscarriage, and spontaneous miscarriage). | | | |
| Study size | 10 | | Explain how the study size was arrived at | | 7 and 9 | | | In this study, all the livebirths in the study area for the period 2016-2018 were included and followed for survival up to the neonatal period. Refusal is rare and the community is always been supportive of icddr,b activities, as they get free treatment for diarrhoea from Dhaka hospital.  During period (2016-2018), there were 6,989 livebirths were registered in the HDSS area and all of them were selected, and of them, 265 died during the neonatal period | | | |
| Quantitative variables | 11 | Explain how quantitative variables were handled in the analyses. If applicable, describe which groupings were chosen and why | | 8 | | First, neonatal mortality was calculated for each week of gestation and for gestation age categories. The relative risk of neonatal death and a 95% confidence interval were calculated using gestational week, while ‘40 weeks’ was used as reference…. | | | |  |  |
| Statistical methods | 12 | (*a*) Describe all statistical methods, including those used to control for confounding | | 7-8 | | The daily death rate up to the neonatal period was calculated with the number of deaths on a given day for the number of new-borns surviving on that day.  The population attributable fraction (percent of deaths that could be prevented if the complications of prematurity could be eliminated) was calculated…..  We performed two both unadjusted (Model-I) and adjusted (Model-II) logistic regression analyses to determine the effect of the gestational age on neonatal mortality. Women’s age at birth, education, working status, sex of the…… | | | |  |  |
|  |  | (*b*) Describe any methods used to examine subgroups and interactions | | NA | |  | | | |  |  |
|  |  | (*c*) Explain how missing data were addressed | | NA | |  | | | |  |  |
|  |  | (*d*) *Cohort study*—If applicable, explain how loss to follow-up was addressed  *Case-control study*—If applicable, explain how matching of cases and controls was addressed  *Cross-sectional study*—If applicable, describe analytical methods taking account of sampling strategy | | 7 | | Refusal is rare and the community is always been supportive of icddr,b activities, as they get free treatment for diarrhoea from Dhaka hospital. | | | |  |  |
|  |  | (*e*) Describe any sensitivity analyses | | NA | |  | | | |  |  |
| Results | | | | | | | | |  |  |  |
| Participants | 13* | (a) Report numbers of individuals at each stage of study—eg numbers potentially eligible, examined for eligibility, confirmed eligible, included in the study, completing follow-up, and analysed | | 9 | | During period (2016-2018), there were 6,989 livebirths were registered in the HDSS area and all of them were selected, and of them, 265 died during the neonatal period (Fig 1). | | | |  |  |
|  |  | (b) Give reasons for non-participation at each stage | | NA | |  | | | |  |  |
|  |  | (c) Consider use of a flow diagram | | 9 and fig 1 | | Fig 1. Flowchart of the sample | | | |  |  |
| Descriptive data | 14* | (a) Give characteristics of study participants (eg demographic, clinical, social) and information on exposures and potential confounders | | 9-10 | | Among them, most of the mother gave birth at 18-24 years of age (54.6%) followed by the 25 or more years (36.7%) and only 8.7% women gave birth at their adolescence…. | | | |  |  |
|  |  | (b) Indicate number of participants with missing data for each variable of interest | | 9 | | During the follow-up period, none of the observations were missing as the culture of the postpartum period in the slum contexts. Women who had live births were usually not moved from their households. | | | |  |  |
|  |  | (c) *Cohort study*—Summarise follow-up time (eg, average and total amount) | | 9 | | The average follow-up period was 27.03 days. | | | |  |  |
| Outcome data | 15* | *Cohort study*—Report numbers of outcome events or summary measures over time | | 14 | | The probability of death for each category of preterm birth as well as those of term births was very high on the day of birth (Figure 1). For very preterm birth, the probability of death on the day of birth was 0.124, 0.048 for moderate preterm, and 0.024 for late preterm; the probability of death was 0.013 for term birth. However, by the 3^rd^ day of life, the probability of death reduced by 70%-95% for these categories of birth. During 7-28 days of life, the probability of death for term birth was 0.002, 0.016 for very preterm birth, 0.020 for moderate preterm birth, and 0.001 for late preterm birth; however, the number of cases for very preterm and late preterm birth were very few. | | | |  |  |
|  |  | *Case-control study—*Report numbers in each exposure category, or summary measures of exposure | |  | |  | | | |  |  |
|  |  | *Cross-sectional study—*Report numbers of outcome events or summary measures | |  | |  | | | |  |  |
| Main results | 16 | (*a*) Give unadjusted estimates and, if applicable, confounder-adjusted estimates and their precision (eg, 95% confidence interval). Make clear which confounders were adjusted for and why they were included | | 14-16 | | Table 4 shows regression estimates of neonatal mortality by preterm birth categories. Model-I included only preterm birth categories, while Model-II included preterm birth categories along with the selected socio-demographic variables as independent variables. For neonatal mortality, Model-I shows that the odds of death was 8.90 (CI: 5.84, 13.57; p<0.01)…. | | | |  |  |
|  |  | (*b*) Report category boundaries when continuous variables were categorized | | 12 | | Both the numbers of births (2.19% and 3.81%) and deaths (12.08% and 10.57%) were low for gestation age categories of 28-31 and 32-33 weeks, but the number of births (15.71%) and the number of deaths (16.98%) increased from gestation age category 34-36 weeks, while both the numbers of births (78.29%) and deaths (60.38%) reached to peak at gestation age category 37-42 weeks | | | |  |  |
|  |  | (*c*) If relevant, consider translating estimates of relative risk into absolute risk for a meaningful time period | | NA | |  | | | |  |  |
| Other analyses | 17 | Report other analyses done—eg analyses of subgroups and interactions, and sensitivity analyses | | NA | |  | | | |  |  |
| Discussion | | | | | | | | |  |  |  |
| Key results | 18 | Summarise key results with reference to study objectives | | 16 | | This study analysed the prospective cohort data for estimating the effects of preterm birth on neonatal mortality in the urban poor (slum) area in Bangladesh. We used two models, where model-I showed the unadjusted effect of preterm birth on neonatal mortality………… | | | |  |  |
| Limitations | 19 | Discuss limitations of the study, taking into account sources of potential bias or imprecision. Discuss both direction and magnitude of any potential bias | | 19 | | The major limitation is the data of conception based on LMP which is criticised for inaccurate recall; however, our record conception may not be a big issue as sufficient probing is done during the data collection and during following visit it is rechecked. | | | |  |  |
| Interpretation | 20 | Give a cautious overall interpretation of results considering objectives, limitations, multiplicity of analyses, results from similar studies, and other relevant evidence | | 16-19 | | Estimation of gestational age based on reported LMP is usually criticised……\|\|… during pregnancy, delivery, and post-partum periods; this might improve survival of the new-borns in general and preterm birth in particular | | | |  |  |
| Generalisability | 21 | Discuss the generalisability (external validity) of the study results | | 18 | | The neonatal mortality rate by gestational age gives us insights into the mortality burden for preterm and term births. For very preterm birth, the neonatal mortality was high, but the proportion of death was low; while for …………….. | | | |  |  |
| Other information | |  | | | | | | | |  |  |
| Funding | 22 | Give the source of funding and the role of the funders for the present study and, if applicable, for the original study on which the present article is based | | 21 | | The study is funded by United Nation’s Children Fund, Bangladesh (Grant number: 01867) | | | |  |  |

NA: Not applicable; NR: Not reported.

*Give information separately for cases and controls in case-control studies and, if applicable, for exposed and unexposed groups in cohort and cross-sectional studies.

**Note:** An Explanation and Elaboration article discusses each checklist item and gives methodological background and published examples of transparent reporting. The STROBE checklist is best used in conjunction with this article (freely available on the Web sites of PLoS Medicine at http://www.plosmedicine.org/, Annals of Internal Medicine at http://www.annals.org/, and Epidemiology at http://www.epidem.com/). Information on the STROBE Initiative is available at www.strobe-statement.org.
