# Supplementary material for: Preterm birth and neonatal mortality in selected slums in and around Dhaka City of Bangladesh: A cohort study
Source: PLoS One. 2024 Jan 19;19(1):e0284005. doi: 10.1371/journal.pone.0284005 (PMC10798464; doi:10.1371/journal.pone.0284005)
Supplement: Attachment — Submitted filename: Review comments_01062023.docx [file pone.0284005.s002.docx]

Manuscript Number: PONE-D-23-08400

**General Comments:**

The study assessed the level and determinants of preterm birth and the contribution of preterm birth to neonatal mortality. I would like to thank the authors for this interesting study and the analysis they conducted. This paper addresses an important topic. I do not have any major criticisms: However, I have some concerns or suggestions that the authors may want to clarify.

**Specific Comments**

1. Did the authors have some pregnancy outcomes in their data that they were not able to register the pregnancies to asses to assess their Last Menstrual Period (LMP) before delivery? If yes, how were they handled?
2. Were there missing values? If yes, how did you handle them?
3. Check and clarify the statement from lines 67-69 “Preterm birth complications are estimated to be responsible for 35% of the world’s 3·1 million annual neonatal deaths, **and are now the second most common cause of death after the economic cost of preterm birth is high in terms of neonate**.”
4. Lines 269-269: The statement “Our study has made an important contribution by proving community-based preterm birth data from the slum dwellers” appears to be wrongly placed. I’m not sure what the intent is. The authors may want to clarify this.
5. Lines 276-277: The statement “Our study indicates that the adolescent women who gave birth to a baby boy by normal delivery had higher risks of neonatal mortality compared to preterm birth” is unclear. The interpretation of the results should be done carefully. Children born to adolescent mothers had increased odds of neonatal death compared to children born to mothers aged 25 or more years. Boys had increased odds of dying as neonates compared to girls, and normal deliveries were associated with increased odds of neonatal death compared to caesarian deliveries. You may want to revise your statement to clarify your statement.
6. Lines 277-279: You may want to clarify or revise the statement “Therefore, raising awareness about late pregnancy and caesarean delivery reduces the risk of neonatal deaths in preterm births.”
