# Supplementary material for: Preterm birth and neonatal mortality in selected slums in and around Dhaka City of Bangladesh: A cohort study
Source: PLoS One. 2024 Jan 19;19(1):e0284005. doi: 10.1371/journal.pone.0284005 (PMC10798464; doi:10.1371/journal.pone.0284005)
Supplement: Attachment — Submitted filename: Response to Reviewers2nd.docx [file pone.0284005.s003.docx]

**Response to Reviewers Comments**

PONE-D-23-08400R1
Preterm Birth and Neonatal Mortality in Selected Slums in and around Dhaka City of Bangladesh: A Cohort Study
We've checked your submission and before we can proceed, we need you to address the following issues:
1. PLOS requires an ORCID iD for the corresponding author in Editorial Manager on papers submitted after December 6th, 2016. Please ensure that you have an ORCID iD and that it is validated in Editorial Manager. To do this, go to ‘Update my Information’ (in the upper left-hand corner of the main menu), and click on the Fetch/Validate link next to the ORCID field. This will take you to the ORCID site and allow you to create a new iD or authenticate a pre-existing iD in Editorial Manager. Please see the following video for instructions on linking an ORCID iD to your Editorial Manager account: <https://www.youtube.com/watch?v=_xcclfuvtxQ>
**Author’s response:** we have added the ORCID.

2. Thank you for stating the following in the Acknowledgments Section of your manuscript:
"icddr,b acknowledges with gratitude the commitment from UNICEF/Sida for continuation of these research efforts. icddr,b is also grateful to the Government of Bangladesh for its long-term financial support and also to international core donors: Canada, Sweden and the United Kingdom."
We note that you have provided funding information that is not currently declared in your Funding Statement. However, funding information should not appear in the Acknowledgments section or other areas of your manuscript. We will only publish funding information present in the Funding Statement section of the online submission form.
Please remove any funding-related text from the manuscript and let us know how you would like to update your Funding Statement. Currently, your Funding Statement reads as follows:

"The study is funded by United Nation’s Children Fund, Bangladesh (Grant number: 01867). The funder played a critical role in preparation of the manuscript."
Please include your amended statements within your cover letter; we will change the online submission form on your behalf.

**Author’s response:** From manuscript we removed funding Statement and added funding statement in cover latter.

3. We note that your Data Availability Statement reads: "Data are available upon request following icddr,b’s data policy. For more details, please visit — https://www.icddrb.org"
Please respond to the below prompts:
A. Did the authors have any special access restrictions other authors would not have?

**Author’s response:** No restrictions at all.

B. Please provide all relevant data set names, variables, instructions, etc. that an independent researcher would need in order to access or request your minimal data set from the link provided.
We will update your Data Availability statement for you upon your response.

**Author’s response:** Data Availability statement included in manuscript.

“Data are available upon request following icddr, b’s data policy. For more details, please visit — <https://www.icddrb.org>**.** However, a subject of birth history and child death data are used and can be requested from Abdur Razzaque, PhD, ([razzaque@icddrb.org](mailto:razzaque@icddrb.org))”
